# Precision Covalent Drug Discovery Inspired by Endogenous Electrophilic Signalling in Immune Cells

**DOI:** 10.3390/biomedicines14081851

**Published:** 2026-08-18

**Authors:** Solomon Habtemariam

**Affiliations:** Pharmacognosy Research & Herbal Analysis Services UK, 124 City Road, London EC1V 2NX, UK; s.habtemariam@herbalanalysis.co.uk

**Keywords:** endogenous electrophilic signalling, precision medicine, immunotherapeutics, immune cell state transition, covalent protein modification, chemoproteomics, immunometabolism

## Abstract

Immune cells possess an intrinsic covalent signalling system in which endogenous electrophilic species function as molecular regulators of cellular state that integrate metabolic activity, oxidative stress, and inflammatory signals. Lipid peroxidation-derived electrophiles, enzymatically generated electrophilic lipid mediators, nitro-fatty acids, and metabolite-derived electrophiles selectively modify reactive amino acid residues within signalling proteins to regulate immune cell differentiation, metabolic adaptation, stress responses, and tissue responses. This perspective highlights that endogenous electrophilic signalling provides a biological blueprint for the development of next-generation precision covalent immunotherapeutics. Beyond exploiting electrophilic chemistry as a reactive pharmacological strategy, emerging insights reveal that immune cells naturally employ regulated covalent modifications to achieve stimulus-dependent control of signalling networks. Advances in chemoproteomics, structural biology, systems immunology, computational chemistry, and targeted delivery technologies are enabling the identification of electrophile-sensitive regulatory targets and the rational design of selective covalent modulators. The discussion includes how the principles derived from endogenous electrophilic signalling can guide therapeutic innovation across major immunotherapeutic areas, including chronic inflammatory diseases, cancer immunology, inflammasome-driven disorders, fibrotic disease, neuroinflammation, and vascular immunometabolic disorders. By translating endogenous covalent regulatory mechanisms into precision drug design strategies, electrophile-guided pharmacology offers an emerging strategy for developing therapies that reprogramme immune cell states, restore immune homeostasis, and achieve stimulus-dependent modulation of disease-associated immune responses.

## 1. Introduction

Designed to protect our body from infection, injury, and cellular stress, inflammation is a fundamental biological process of immune homeostasis orchestrated by coordinated interactions between immune cells, stromal components, and tissue-derived signals. Excessive and dysregulated inflammatory responses, however, transition these protective mechanisms into pathological pathways of diseases such as autoimmune, metabolic, cardiovascular, fibrotic, and neurodegenerative disorders. As a result, therapeutic modulation of immune responses has become the principal objective of modern drug discovery.

For several decades, therapeutic approaches for immune-mediated diseases have largely relied on targeting defined components of inflammatory pathways, including extracellular enzymes, cytokine networks, and intracellular signalling cascades. Biological agents directed against tumour necrosis factor (TNF), interleukin (IL)-1, and IL-6 [1], along with small-molecule inhibitors targeting kinase pathways such as Janus kinase-signal transducer and activator of transcription (JAK-STAT) signalling [2], have transformed the clinical management of chronic inflammatory disorders. Anti-cytokine therapies primarily function through ligand sequestration or receptor blockade, leading to interruption of extracellular communication pathways. On the other hand, small-molecule kinase inhibitors [3,4] modulate signalling intensity through occupancy-dependent, mostly reversible inhibition of enzymatic activity. Although these approaches have produced substantial clinical benefits, they generally intervene at predefined signalling targets without directly addressing the molecular processes that determine immune cell state transitions, adaptation, and resolution. As a consequence, many therapies suppress inflammatory activities without necessarily restoring the endogenous regulatory mechanisms that coordinate immune homeostasis, tissue repair, and functional immune balance.

Emerging evidence suggests that immune regulation is not exclusively controlled through receptor-mediated signalling pathways. Alternative regulatory pathways include chemically encoded mechanisms involving reactive endogenous metabolites and lipid-derived signalling molecules. During immune activation, particularly in macrophages, neutrophils, and inflamed tissue microenvironments, cells generate diverse electrophilic species arising from lipid peroxidation, enzymatic oxidation of polyunsaturated fatty acids, nitric oxide (NO)-reactive oxygen species (ROS) chemistry, and metabolite-derived transformations associated with altered immune metabolism. Traditionally, these molecules were largely viewed as indicators or markers of oxidative damage or cellular stress. However, accumulating evidence demonstrates that selected electrophilic species act as regulated signalling mediators capable of modifying immune cell function through specific molecular interactions [5,6]. A prominent feature of endogenous electrophilic molecules is their capacity to undergo covalent modification of nucleophilic amino acid residues, particularly cysteine thiols, within proteins involved in cellular signalling and metabolic regulation [7,8]. These covalent modifications can alter transcriptional responses, metabolic programmes, stress adaptation pathways, and inflammatory phenotypes. Hence, far more than mere consequences of nonspecific chemical reactivity, such interactions represent regulated events biased toward redox-sensitive regulatory proteins that function as molecular control gates within immune signalling networks.

The article herein focuses on the emerging concept of endogenous electrophilic signalling that describes a chemically encoded mechanism of immune regulation which operates alongside classical receptor-mediated, transcriptional, and metabolic signalling pathways. By acting as reversible or state-dependent molecular switches, electrophile-sensitive signalling networks may provide immune cells with a mechanism to integrate environmental stress, metabolic status, and inflammatory activation. Understanding these endogenous covalent regulatory processes offers a new opportunity for drug discovery whereby, beyond simply blocking individual inflammatory pathways, therapeutic strategies may be designed to mimic, enhance, or redirect endogenous electrophilic signalling to achieve precision modulation of immune cell states.

## 2. Endogenous Electrophilic Signalling in Immune Cells

**Figure 1:**
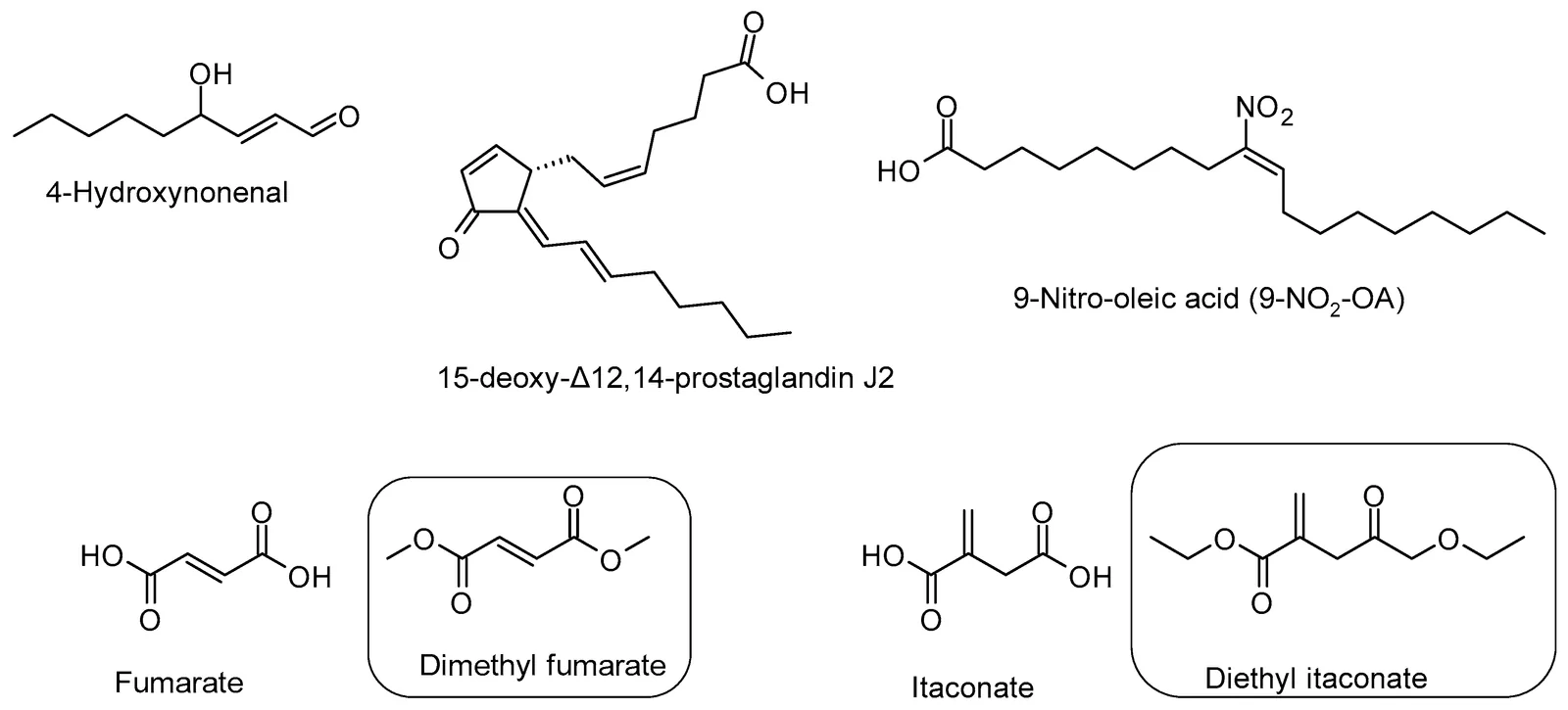
Examples of endogenous electrophiles produced in immune cells. Structures in boxes represent examples of experimental drugs derived from these endogenous emetophiles. Only a selected few are presented. For example, nitro oleic acid can be represented as 9-nitro or 10-nitro derivatives, while polyunsaturated fatty acids (e.g., linoleic acid) can have this nitro functional group located at various sites.

Immune activation is accompanied by profound changes in cellular redox balance, lipid metabolism, and mitochondrial function, all of which are involved in the generation of chemically reactive electrophilic small molecules. As discussed in the following sections, a growing body of evidence indicates that many of these species are produced in a temporally and spatially regulated manner, particularly within activated immune cells, and inflamed tissue microenvironments. These electrophilic molecules are capable of covalent modification of nucleophilic residues in proteins, thereby exerting direct control over immune signalling networks. A central feature of this system is that electrophilic signalling arises from multiple convergent sources that span lipid oxidation chemistry, enzymatic lipid mediator biosynthesis, NO-ROS interactions, and metabolite-driven electrophilic transformations (Figure 1). Specific examples used for this discussion include lipid peroxidation during oxidative stress, which generates reactive aldehydes such as 4-hydroxynonenal (4HNE) [8], which can covalently modify proteins involved in inflammatory signalling and redox sensing. Enzymatic lipid oxidation pathways produce cyclopentenone prostaglandins, including 15-deoxy-Δ12,14-prostaglandin J2 (15d-PGJ2), which function as electrophilic mediators capable of modulating key inflammatory transcription factors such as nuclear factor kappa-light-chain-enhancer of activated B cells (NF-κB) and activating cytoprotective signalling through the Keap1-Nrf2 (Kelch-like ECH-associated protein 1 - nuclear factor erythroid 2-related factor 2) axis [9,10]. At the same time, NO and lipid radical chemistry generate nitrated fatty acids (NO_2_-FAs), including nitro-oleic acid (NO_2_-OA), which reversibly alkylate signalling proteins and modulate inflammatory responses through covalent yet dynamically reversible interactions [11]. In addition, metabolite-derived electrophilic species, most notably those associated with fumarate chemistry and itaconate-derived derivatives, have emerged as important regulators of redox-sensitive signalling networks in immune cells [12]. Despite their diversity, these systems share a unifying functional outcome in which the generation of reactive chemical species modulates immune-state transitions through covalent protein modification.

### 2.1. Lipid Peroxidation-Derived Electrophiles as Signalling Molecules

**Figure 2:**
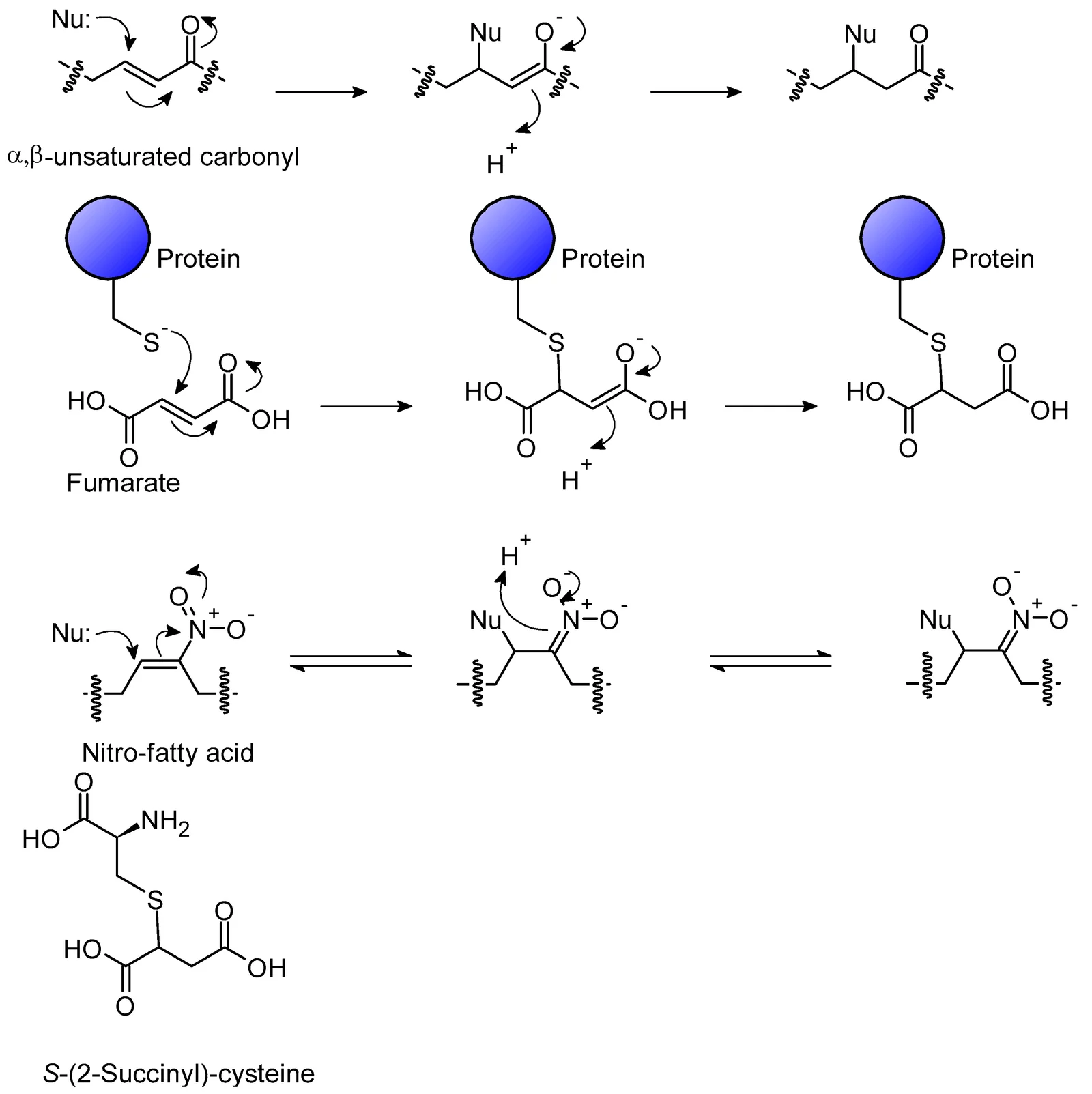
Michael addition reaction involving endogenously produced electrophiles. Covalent modification of proteins by functional groups possessing an α,β-unsaturated moiety via the sensitive cysteine amino acid residue is shown. The thiol (SH) group of cysteine serves as an electron-rich nucleophile (Nu:). In the case of fumarate, *S*-(2-succinyl)-cysteine is formed through this mechanism, which is often called the succination reaction. Strong electron-withdrawing nitro groups in nitro-fatty acids (nitro-alkenes) also lead to similar covalent modification, but this reaction is reversible.

One of the most extensively characterised sources of electrophilic signalling molecules in immune biology is lipid peroxidation. During inflammatory activation, particularly in macrophages and neutrophils undergoing respiratory burst, ROS induce oxidation of polyunsaturated fatty acids within cellular membranes [12]. This process generates a range of reactive aldehydes and electrophilic lipid fragments, including 4HNE, malondialdehyde, and acrolein. Although these products are largely viewed as markers of oxidative damage, they exhibit selective reactivity toward protein thiols and other nucleophilic residues. 4-Hydroxynonenal, in particular, has been shown to form Michael adducts (Figure 2) with signalling proteins involved in redox sensing and inflammatory regulation [13]. The thiol (-SH) group of a cysteine residue, as a potent nucleophile, can attack the electrophilic C3 carbon of 4HNE’s α,β-unsaturated double bond, leading to adduct formation [14]. While this reaction mechanism can be applied to many amino acids, cysteine is by far the most sensitive amino acid known in this reaction, followed by histidine and then lysine [15]. This reaction mechanism is also no longer seen as an indiscriminate means of modifying proteins as it appears to act as a potent secondary messenger of oxidative stress with effects biased toward redox-sensitive regulatory targets [16], including components of NF-κB, Nrf2, mitogen-activated protein kinases (MAPK), toll-like receptor-4 (TLR4), and stimulator of interferon genes (STING) signalling and antioxidant defence pathways in immune cells [17]. Moreover, the biological effects of these molecules are concentration- and pathology-dependent. At high levels, they contribute to cellular stress and tissue damage, whereas at lower physiologically relevant concentrations, they can modulate signalling pathways and transcriptional programs [17,18]. This dual effect suggests that lipid peroxidation products occupy a spectrum between metabolic byproducts and functional signalling mediators.

### 2.2. Enzymatically Generated Electrophilic Lipid Mediators Serve as Regulated Covalent Signalling Molecules

In contrast to lipid peroxidation products, certain electrophilic species are generated through tightly regulated enzymatic pathways, particularly within the arachidonic acid cascade. A prominent example is the class of cyclopentenone prostaglandins, including 15d-PGJ2. These molecules contain highly reactive α,β-unsaturated carbonyl groups that act as Michael acceptors capable of covalent modification of cysteine residues in signalling proteins [19]. They belong to the cyclopentenone prostaglandins, and unlike non-enzymatic oxidation products, they are produced in a temporally regulated manner during inflammatory resolution phases, suggesting a role in actively shaping the transition from inflammation to homeostasis. Mechanistically, 15d-PGJ2 has been shown to modify Keap1 through covalent adduct formation [10,20], thereby stabilising Nrf2 and inducing transcriptional programs associated with antioxidant defence and anti-inflammatory signalling. In addition, it can interfere with NF-κB signalling through modification of pathway components, thereby suppressing pro-inflammatory gene expression. Direct inhibition and modification of the IKKβ subunit of IKK, leading to inhibition of activation of NF-κB by pro-inflammatory stimuli, has been described as an established mechanism for these compounds [21]. Therefore, this class of compounds represents one of the clearest examples of endogenous electrophilic signalling in mammalian biology to demonstrate that enzymatically controlled lipid-derived electrophiles can function as significant regulators of immune-state transitions.

### 2.3. Nitro-Fatty Acids Serve as Reversible Electrophilic Signalling in Inflammatory Environments

A further class of electrophilic mediators arises from the interaction of NO-derived species with unsaturated lipids, resulting in the formation of nitrated fatty acids such as NO_2_-OA. These compounds possess electrophilic nitroalkene groups that undergo reversible Michael addition with protein thiols (as strong nucleophiles), leading to covalent modification of signalling proteins [22]. Consequently, the electron-poor β-carbon next to the strongly electron-withdrawing nitro group enables a reversible covalent bond (Figure 2) to form with nucleophilic targets inside the cell. When these specific protein thiols are alkylated, primarily transcription factors like NF-κB, it can alter subsequent gene activation, leading to changes in associated metabolic and inflammatory processes. Thus, NO_2_-fatty acids have been shown to modulate multiple inflammatory signalling pathways, including NF-κB and peroxisome proliferator-activated receptor-γ (PPARγ)-dependent transcriptional programs [23]. For instance, altering IκB kinase β (IKKβ) disrupts its normal phosphorylation cascade, and this aberration prevents the phosphorylation of the inhibitor of κB (IκBα), which halts its subsequent ubiquitination and proteasomal degradation. By leaving this inhibitor intact to continually sequester the transcription factor in the cytoplasm, the disruption ultimately blocks NF-κB activation. We should note that this reversible covalent modification distinguishes NO_2_-FAs from irreversible electrophiles (permanent protein inactivation), allowing them to function as transient modulators of regulatory protein activity, and enabling fine-tuning of immune responses. Hence, unlike 4HNE, which forms highly stable and/or permanent adducts, the NO_2_-OA-cysteine Michael adduct is shown to be kinetically fast but thermodynamically reversible [24]. The adduct can readily dissociate to regenerate the free fatty acid and the native cysteine residue.

Not surprisingly, nitroalkenes downregulate the expression and/or activity of pro-inflammatory signalling molecules induced by various stimuli, including IL-6, LPS, TGF-β, and TNF-α. While inhibiting NF-κB, NO_2_-FAs also simultaneously activate the nuclear factor Nrf2, leading to activation of an antioxidant signalling pathway [25]. Accordingly, NO_2_-FAs exhibit inhibitory effects on the activation of human neutrophils, as well as on superoxide generation, degranulation, and CD11b integrin adhesion molecule expression [26]. Experimental evidence also suggests that in vivo treatment with NO_2_-OA, which maintains nanomolar plasma concentrations, was sufficient to inhibit lipopolysaccharide (LPS)-induced TLR4 cell surface expression in leukocytes and NF-κB activation [27,28]. In the same study, in vitro, NO_2_-OA suppresses LPS-induced TLR4 signalling by inhibiting IκBα phosphorylation and impairing the recruitment of TLR4 and TNF receptor-associated factor-6 to lipid raft compartments. From a pharmacological perspective, NO_2_-FAs represent a unique class of endogenous electrophiles as they demonstrate covalent modification of signalling proteins which can be both reversible and functionally beneficial as immunomodulators.

### 2.4. Metabolite-Derived Electrophiles as a Link Between Immunometabolism and Covalent Signalling

Recent advances in immunometabolism have revealed that metabolic intermediates can also give rise to electrophilic signalling species. The most common example is fumarate, a tricarboxylic acid cycle intermediate which can undergo a Michael addition reaction with cysteine residues in certain proteins, leading to the production of *S*-(2-succinyl)-cysteine (Figure 2). The reaction is called cysteine succination. Fumarate hydratase deficiency in cancer and diabetes is among the pathologies where accumulation of fumarate has been observed [29,30]. Fumarate-derived electrophilic species can modify cysteine residues in proteins, including those involved in redox sensing. High intracellular levels of fumarate cause irreversible succination of Keap1, leading to Nrf2 signalling [31]. Thus, dimethyl fumarate, as an orally active small-molecule drug approved for multiple sclerosis and psoriasis, exerts its therapeutic effects in part through covalent modification of Keap1 cysteine residues [32]. This leads to activation of Nrf2-dependent cytoprotective and anti-inflammatory gene activations, representing a clinically validated example of electrophile-based immunomodulation.

Similarly, itaconate (Figure 1) and its derivatives, such as dimethyl itaconate and 4-octyl itaconate, have emerged as important regulators of inflammatory signalling in macrophages. These metabolites possess electrophilic properties that enable modification of redox-sensitive proteins, leading to suppression of pro-inflammatory cytokine production and promotion of anti-inflammatory states. They have been shown to display an anti-inflammatory property through modifying cysteine residues within protein targets, leading to production of eosinophil-targeted chemokines in a variety of cell types, including M1 and M2 macrophages, Th2 cells, and A549 respiratory epithelial cells [33]. Hence, fumarate- and itaconate-derived electrophiles provide a mechanistic bridge between immunometabolism and covalent signalling. They demonstrate that metabolic reprogramming in immune cells can directly generate electrophilic species with signalling capacity. In addition to covalent modification of proteins, inhibition of succinate dehydrogenase, epigenetic modification, and modulation of G-protein-coupled receptor oxoglutarate receptor 1 have also been reported [34]. Using itaconate as a model compound, metabolic control of neuroinflammation, such as central nervous system (CNS) disorders, is also emerging as a therapeutic option [35].

Overall, endogenous electrophilic mediators converge on key immune regulatory hubs, including the Keap1-Nrf2 pathway, NF-κB signalling, inflammasome proteins, and metabolic enzymes, where covalent protein modification regulates inflammatory and antioxidant responses. Their therapeutic relevance is exemplified by dimethyl fumarate, an approved treatment for multiple sclerosis and psoriasis that activates Nrf2 through Keap1 modification, while NO_2_-FAs are also being explored as anti-inflammatory agents. Despite this translational success, endogenous electrophilic signalling remains fragmented across redox biology, lipid signalling, and immunometabolism, as they are not recognised as a central mechanism of immune regulation. These evolutionarily conserved electrophilic signals, however, seem to provide a biological blueprint for covalent anti-inflammatory drug design, enabling state-selective immunomodulation by harnessing the same chemical signalling principles used by immune cells. This approach is broadly in line with metabolic reprogramming and immunometabolism as a base for novel therapeutic discovery [36,37].

## 3. A Blueprint for Covalent Drug Design Guided by Endogenous Electrophilic Signalling in Immune Cells

**Figure 3:**
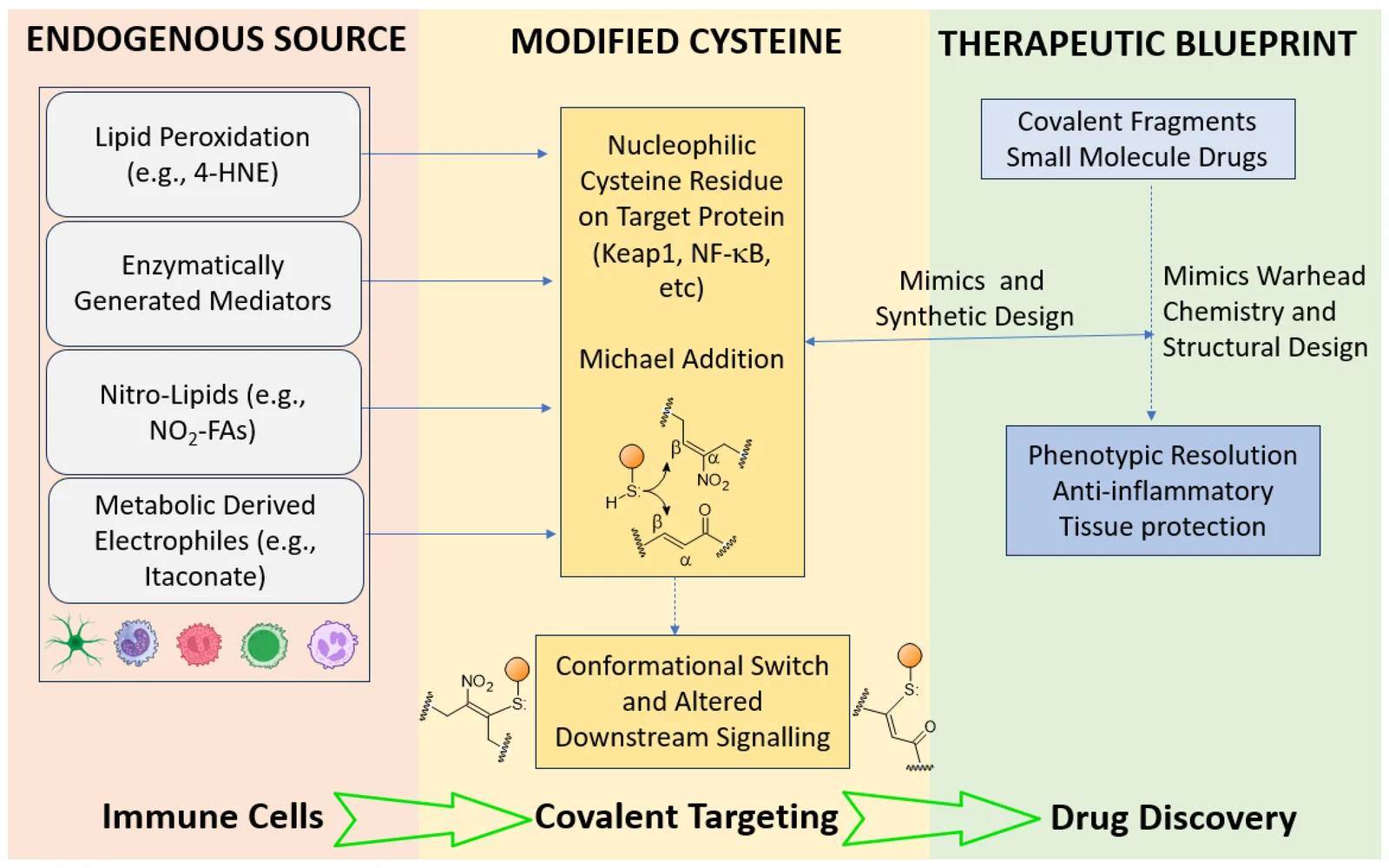
Translating endogenous electrophilic signalling into covalent blueprints. The figure depicts how endogenous electrophilic stress signals provide chemical principles that can be translated into rational covalent drug design. (**Left**): cellular stress generates endogenous electrophiles, including lipid- and metabolite-derived electrophilic species. (**Middle**): these electrophiles covalently modify nucleophilic residues, particularly cysteine thiols, in regulatory proteins such as KEAP1 and components of NF-κB signalling, thereby inducing conformational or functional changes that alter downstream signalling. (**Right**): medicinal chemistry can exploit this endogenous electrophile–nucleophile chemistry by using endogenous electrophiles as chemical templates or inspiration for synthetic mimics and by incorporating appropriately tuned electrophilic warheads into molecular scaffolds that provide molecular recognition and positioning for selective covalent target engagement. This translation from endogenous chemical signalling to controlled covalent modification provides a blueprint for precision covalent drug discovery and the development of therapeutic leads. 4-HNE, 4-hydroxynonenal; NO_2_-FAs, nitro-fatty acids; Keap1, Kelch-like ECH-associated protein 1; NF-κB, nuclear factor kappa B.

The discovery that immune cells utilise endogenous electrophilic molecules as regulatory signals has introduced a new dimension to therapeutic intervention. Beyond the classical inflammatory pathways that rely predominantly on receptor–ligand interactions, electrophilic signalling operates through selective covalent modification of nucleophilic residues, particularly cysteine residues within regulatory proteins. These modifications function as molecular switches that integrate metabolic status, oxidative stress, and inflammatory activation. The physiological existence of such regulated covalent signalling suggests that the immune system itself provides a blueprint for the development of next-generation covalent drugs. Thus, future drug discovery strategies may exploit endogenous electrophilic mechanisms to achieve microenvironment-dependent modulation of immune cell function (Figure 3).

### 3.1. Chronic Inflammatory Diseases: Restoring Immune Homeostasis Through Electrophilic Pathway Modulation

Chronic inflammatory disorders present a major opportunity for exploiting endogenous electrophilic signalling as persistent immune activation is frequently associated with disruption of metabolic and redox regulatory networks. As stated above, current therapeutic approaches primarily target extracellular inflammatory mediators, including cytokines and their receptors, where interventions often globally suppress inflammation, which may compromise protective immune responses. Endogenous electrophilic pathways offer an alternative strategy by regulating intracellular immune cell states rather than blocking individual inflammatory outputs. Metabolically derived electrophiles, particularly itaconate and related electrophilic intermediates generated during macrophage activation, demonstrate how immune cells naturally establish negative feedback mechanisms to limit excessive inflammation. Through covalent modification of regulatory proteins, these molecules influence pathways including Nrf2 activation, NF-κB signalling, inflammasome activity, and inflammatory metabolism. This provides a principal approach for developing covalent therapeutics that mimic or enhance endogenous resolution pathways. An interesting insight into this dual targeting approach in inflammation modulation through electro functional groups is reviewed by O’Brien and Wendell [5]. Such approaches may offer far better outcomes than producing general immunosuppression, as it could potentially restore immune equilibrium by reprogramming inflammatory macrophages and myeloid cells.

### 3.2. Cancer Immunotherapy: Exploiting Electrophilic Signalling to Reshape Tumour-Associated Immune States

The tumour microenvironment represents a unique biological setting in which electrophilic signalling may be therapeutically exploited. Tumours generate high levels of oxidative stress, lipid peroxidation products, and metabolic intermediates that profoundly influence immune cell behaviour [38]. Tumour-associated macrophages and myeloid-derived suppressor cells adapt to this chemically altered environment, often acquiring immunosuppressive phenotypes that support tumour growth and resistance to therapy [39]. Understanding how endogenous electrophiles regulate these immune adaptations creates opportunities for a new class of covalent immunomodulators. Beyond targeting tumour cells directly, electrophile-inspired covalent drugs could be designed to modify immune cell signalling networks and restore anti-tumour immunity. Modulation of pathways involving Keap1-Nrf2, inflammatory metabolism, and redox-sensitive immune checkpoints may provide strategies to convert tumour-supportive myeloid populations into immune-stimulatory states [40].

### 3.3. Inflammasome-Driven Disorders: Targeting Endogenous Electrophilic Checkpoints of Innate Immunity

The inflammasome represents another therapeutic area where endogenous electrophilic signalling provides a mechanistic framework for covalent drug discovery. Inflammasome activation is tightly linked to mitochondrial dysfunction, oxidative stress, and metabolic disturbance [41], all of which generate electrophilic species capable of modifying regulatory proteins. These modifications act as intrinsic control mechanisms that prevent uncontrolled inflammatory activation. The success of direct inflammasome inhibitors has demonstrated the therapeutic value of targeting innate immune activation [42]. However, covalent modulation inspired by endogenous electrophilic regulation could offer an alternative strategy by targeting regulatory targets that control inflammasome activity instead of simply blocking downstream activation. Reversible covalent targeting approaches may be particularly attractive as they could provide sustained pathway modulation while reducing the risks associated with permanent protein inhibition. The proof of concept has already been demonstrated by endogenous metabolites like 4HNE [42]. The challenge and opportunity lie in identifying regulatory cysteines that function as biological control points within inflammasome networks.

### 3.4. Fibrotic Disease: Manipulating Immune-Stromal Communication Through Electrophilic Regulation

Fibrosis is another pathological state in which normal tissue repair mechanisms become chronically activated. Persistent macrophage activation, altered endothelial responses, and fibroblast stimulation create a self-reinforcing environment characterised by oxidative stress and metabolic dysfunction [43]. Endogenous electrophilic signalling provides an important regulatory interface between these processes by controlling immune cell phenotype, redox adaptation, and tissue remodelling responses. A major therapeutic challenge in fibrosis is achieving suppression of pathological repair without impairing normal regeneration. Covalent drugs designed around endogenous electrophilic mechanisms may be a selective avenue in restoring tissue homeostasis. By modulating immune metabolic pathways, macrophage activation states, and stress response networks, such therapies could direct the trajectory of inflammation-driven repair toward resolution. With this approach, we are moving our effort away from targeting fibrosis as an endpoint to correcting the immune signalling abnormalities that initiate and sustain pathological remodelling.

### 3.5. Neuroinflammation: Leveraging Electrophilic Signalling to Restore Immune Equilibrium in the Nervous System

The CNS is another of the best examples for the application of endogenous electrophilic signalling, as microglia and other immune-associated cells are highly dependent on metabolic adaptation. Chronic activation of these cells contributes to neurodegenerative pathology, but complete suppression of immune function is still undesirable as immune surveillance and repair mechanisms remain essential. Electrophilic pathways regulate multiple processes relevant to neuroinflammation, including mitochondrial function, oxidative stress responses, inflammasome activation, and inflammatory phenotype switching. For example, the Nrf2-Keap1 signalling axis mitigating neuroinflammation by boosting antioxidant defences, preserving mitochondrial respiration, restricting inflammasome assembly, and promoting the restorative M2 over the pro-inflammatory M1 microglial phenotype, has been reviewed for Parkinson’s disease [44]. Recent literature also advocated the potential of itaconate in regulating the functional state of microglia by inhibiting NF-κB signalling, succinate dehydrogenase synthesis and NLRP3 inflammatory vesicle activation, while promoting the activation of the Nrf2 signalling pathway [35,45]. Harnessing these mechanisms could enable the development of covalent immunomodulators that promote protective immune states. The development of CNS-accessible selective covalent modulators remains challenging, but the underlying biology provides a strong rationale for exploring electrophilic pharmacology in neuroimmune disorders. Targeting neurodegenerative diseases through kinase-based covalent inhibitors has also been similarly advocated [46].

### 3.6. Vascular Inflammation and Immunometabolic Disease: Targeting the Immune–Vascular Interface

The immune–vascular interface provides an additional therapeutic option in which endogenous electrophilic signalling may guide drug discovery. Vascular inflammation, endothelial dysfunction, and pathological remodelling are strongly influenced by immune cell activation, lipid metabolism, oxidative stress, and inflammatory signalling [47]. Electrophilic lipid mediators and metabolic electrophiles represent important regulators of these processes by modifying signalling proteins involved in inflammation, endothelial adaptation, and tissue repair. Beyond angiogenic growth factors or inflammatory cytokines, endogenous electrophilic signalling highlights the importance of immune-metabolic regulation in vascular biology. Covalent drugs inspired by these pathways may provide opportunities to promote vascular repair while limiting chronic inflammatory activation. Such approaches could have relevance across atherosclerosis, diabetes-associated vascular complications, and ischemic tissue injury.

The above-mentioned six therapeutic areas altogether exemplify the emerging concept that endogenous electrophilic signalling represents a naturally evolved system of covalent immune regulation. Furthermore, this concept already has translational precedents as exemplified by some small molecular leads. Dimethyl fumarate (Figure 1), a fumarate-derived electrophile approved for multiple sclerosis, covalently modifies reactive cysteine residues in Keap1 and activates Nrf2 signalling [48,49]. Bardoxolone methyl, an electrophilic triterpenoid that reached advanced clinical development, provides a further example of pharmacological Nrf2 activation through covalent engagement of Kea1 Cys151 [50,51]. Omaveloxolone, a reversible electrophilic Nrf2 activator that targets Keap1 Cys151, has progressed to clinical use for Friedreich ataxia [52,53]. In addition, sulforaphane, a naturally occurring electrophile that modifies Keap1 cysteines and activates Nrf2, has provided extensive preclinical and clinical proof-of-principle for pharmacological exploitation of this endogenous sensing pathway [54]. These examples demonstrate that the therapeutic translation of electrophilic signalling is already feasible, while also highlighting the opportunity to extend this principle to less explored immune-regulatory pathways and disease settings. The objective of future covalent drug discovery is therefore not simply to introduce reactive chemical groups into therapeutic molecules, but to understand and reproduce the principles by which immune cells use covalent chemistry to maintain functional balance.

## 4. Future Perspectives and Translational Opportunities

**Figure 4:**
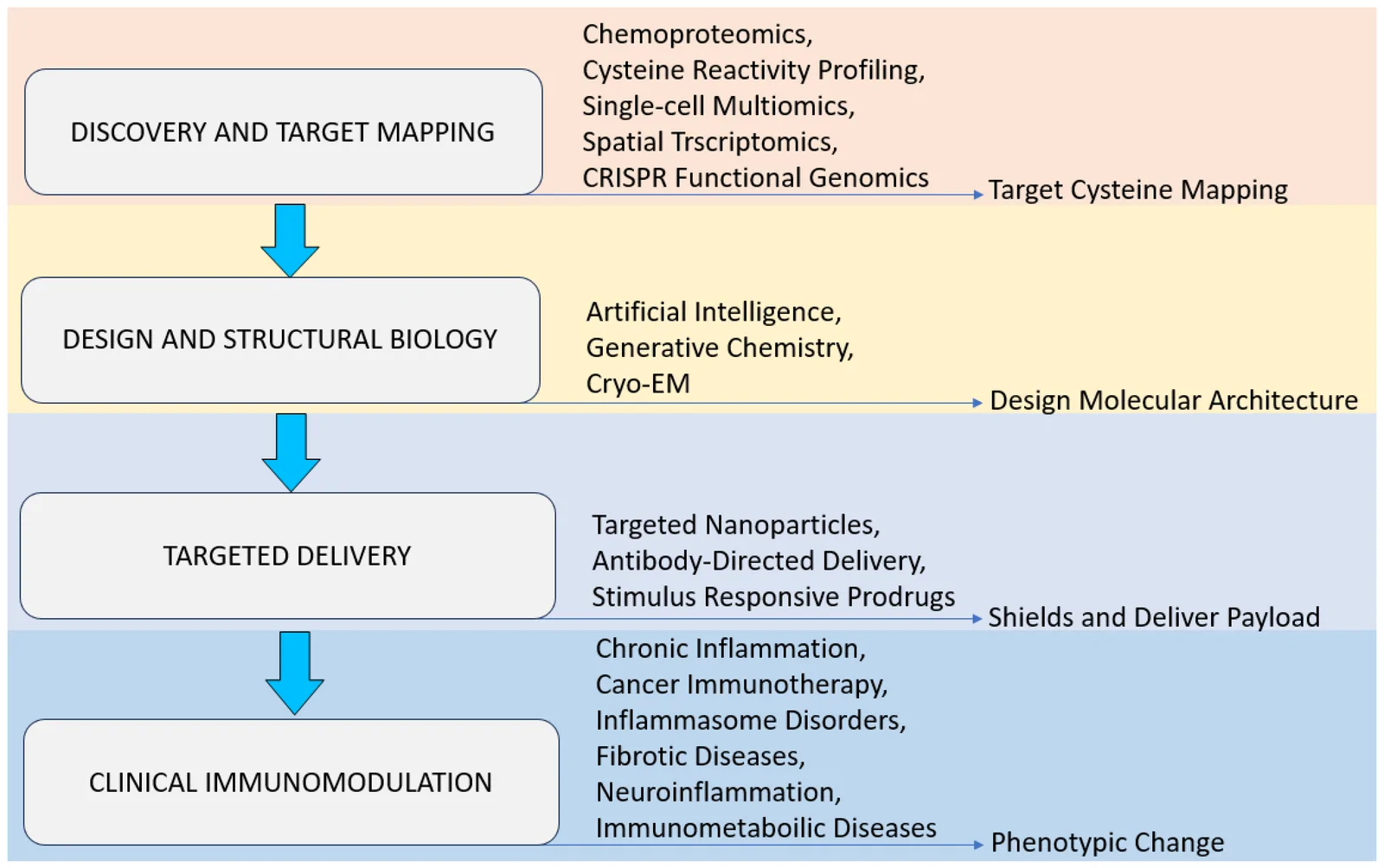
Technology pipeline for translating endogenous electrophilic signalling into targeted covalent therapies. The figure outlines a prospective technology-enabled pipeline for translating electrophile-sensitive signalling targets into therapeutic candidates. Discovery and target mapping: chemoproteomics, cysteine reactivity profiling, single-cell multi-omics, spatial transcriptomics and CRISPR-based functional genomics can be integrated to identify and prioritise therapeutically accessible and biologically relevant cysteine targets within immune cell signalling networks. Drug design: artificial intelligence (AI), generative chemistry and structural biology, including cryo-electron microscopy (cryo-EM), can support the design and optimisation of covalent small molecules by integrating target structure, molecular recognition and electrophile reactivity. Targeted delivery: targeted nanoparticles, antibody-directed delivery and stimulus-responsive prodrugs may subsequently be used to improve delivery to selected immune cell populations and enhance therapeutic selectivity. Translational application: these technologies collectively provide a prospective route towards therapeutic immunomodulation across the disease areas discussed in this perspective, including chronic inflammation, cancer, inflammasome-driven diseases, fibrosis, neuroinflammation and vascular inflammation. The pipeline is intended as a forward-looking framework for integrating emerging technologies to refine target identification, covalent drug design and targeted delivery, rather than implying that all components have already been validated as a unified clinical workflow. CRISPR, clustered regularly interspaced short palindromic repeats.

Despite its considerable therapeutic promise, electrophile-guided covalent immunomodulation remains in its infancy. A major challenge lies in balancing the intrinsic reactivity of electrophilic molecules with the selectivity required for safe therapeutic intervention. Excessive or uncontrolled electrophilic reactivity can result in off-target protein modification and cytotoxicity, whereas endogenous electrophiles operate within tightly regulated spatial and temporal attributes that ensure selective modulation of immune signalling. Replicating these characteristics through synthetic drug design will require careful optimisation of electrophile reactivity, target selectivity and pharmacokinetic properties. Additional challenges include the incomplete identification of physiologically relevant electrophile-sensitive proteins, distinguishing adaptive electrophilic signalling from oxidative damage, and understanding the temporal dynamics of covalent protein modification during immune activation and resolution. Although these limitations have constrained therapeutic translation, they are being addressed through advances in biotechnology and biopharmaceutical sciences (Figure 4).

Among the most transformative developments in the electrophile-based therapeutic field is the emergence of chemoproteomics [55], which has shifted covalent drug discovery from empirical screening towards systematic mapping of accessible amino acid residues across the proteome. Activity-based protein profiling and quantitative cysteine reactivity profiling [56,57] now enable identification of electrophile-sensitive proteins within specific immune cell populations under defined inflammatory conditions. This allows regulatory cysteine residues that function as signalling switches to be distinguished from nonspecific oxidative modifications. Integration with single-cell multi-omics, spatial transcriptomics and CRISPR-based functional genomics [58,59,60] further provides insight into how electrophilic signalling varies between immune cell subsets, tissues and disease microenvironments, facilitating identification of disease-specific electrophile-responsive pathways while preserving physiological immune function.

Based on these new technological advances, future therapeutics (Figure 4) can be designed to emulate key features of endogenous electrophilic signalling, including reversible covalent interactions, kinetic selectivity and preferential modification of regulatory cysteine residues. Such strategies offer the potential to selectively restore endogenous pathways that promote the resolution of inflammation without indiscriminately suppressing immune responses, thereby improving both efficacy and safety. The innovations in pharmaceutical development can further address long-standing limitations associated with electrophilic therapeutics. Nanoparticle-based formulations, lipid nanoparticles, antibody-directed delivery systems and stimulus-responsive prodrugs provide opportunities to restrict electrophilic agents to inflamed tissues or selected immune cell populations, reducing systemic exposure and limiting off-target protein modification. This is the same pharmacokinetics principle that is already routinely being applied in various disease therapeutic approaches, including in cancer, inflammatory diseases and CNS disorders. At the same time, advances in artificial intelligence (AI), structural biology and computational drug design are accelerating the identification of ligandable residues, prediction of covalent binding kinetics and optimisation of electrophilic scaffolds [61,62]. Altogether, these complementary technologies are transforming endogenous electrophilic signalling from a mechanistic concept into a continuously evolving framework for therapeutic development. Thus, future progress will largely depend on integrating chemical biology, systems immunology, biotechnology and pharmaceutical engineering to exploit endogenous electrophilic signalling with greater precision (Figure 4). As our understanding of electrophile-sensitive signalling networks continues to expand, these interdisciplinary approaches are expected to facilitate the development of safer, more selective covalent immunomodulators capable of restoring immune homeostasis while minimising the adverse effects associated with conventional anti-inflammatory therapies.

## 5. Conclusions

Endogenous electrophilic signalling represents a chemically encoded mechanism of immune regulation that offers a compelling blueprint for next-generation covalent immunomodulatory drug discovery. These mediators should no longer be viewed as byproducts of oxidative stress, but as endogenous electrophiles capable of orchestrating immune state transitions through selective covalent modification of redox-sensitive signalling networks. The endogenous electrophilic signalling further provides principles for designing therapeutics that restore immune function beyond suppressing it. Recent advances in chemoproteomics, systems immunology, AI, and targeted drug delivery now provide unprecedented opportunities to identify electrophile-responsive targets, optimise covalent ligands, and translate these endogenous regulatory mechanisms into precision therapeutics. As biotechnology and pharmaceutical innovation continue to converge, harnessing the design principles of endogenous electrophilic signalling may establish a new paradigm for precision covalent immunomodulation and the treatment of many diseases.

## Data Availability

No new data were created or analysed in this study. Data sharing is not applicable to this article.
